# Supplementary material for: Spheroid-Like Cultures for Expanding Angiopoietin Receptor-1 (aka. Tie2) Positive Cells from the Human Intervertebral Disc
Source: Int J Mol Sci. 2020 Dec 10;21(24):9423. doi: 10.3390/ijms21249423 (PMC7763454; doi:10.3390/ijms21249423)
Supplement: Supplementary file 1 [file ijms-21-09423-s001.pdf]

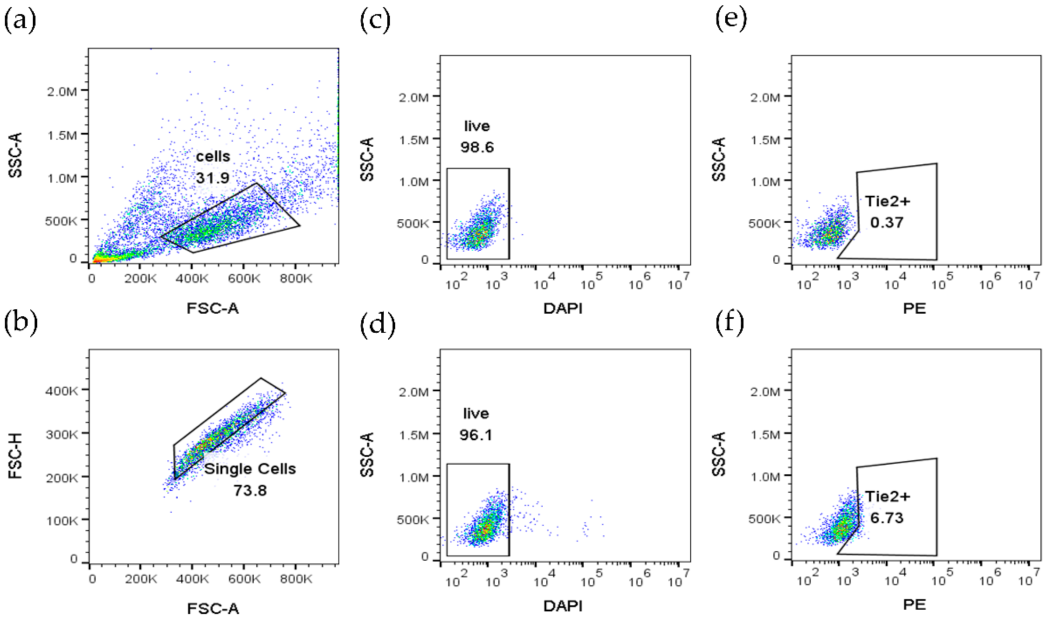

Supplementary Figure S1. Flow cytometric isolation of Tie2+NPC (a) Exclusion of debris on a plot of SSC-A (side scatter area) vs FSC-H (forward scatter height). (b) Gating on single cells and excluding clumps on a plot of FSC-A (forward scatter area) vs FSC-H (forward scatter height). (c) Unstained control with DAPI exclusion gate on a plot of SSC-A vs DAPI. "Live" represents DAPI exclusion gate. (d) Gating for viable cells by DAPI exclusion on a plot of SSC-A vs DAPI. (e) Isotype control with Tie2+ gate on a plot of SSC-A vs PE (f) Sample stained with PE-conjugated anti-Tie2 antibody gated for Tie+ cells on a plot of SSC-A vs PE.

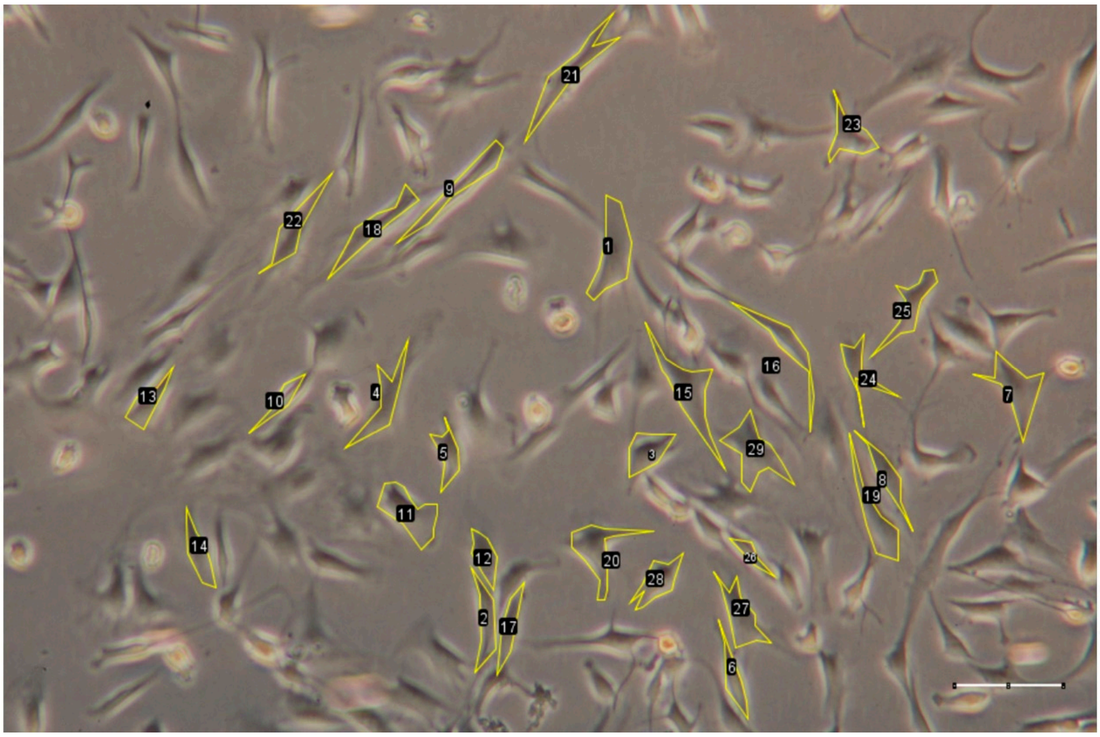

Supplementary Figure S2. A representative image of the cell shapes of the NPCs cultured on "classic"surface. The segmentation of the cells was defined manually and the morphology of cells was then quantified by ImageJ v1.53c. Scale bar = 100 μm.
